# Vitamin D metabolism and extraskeletal outcomes: an update

**DOI:** 10.20945/2359-3997000000565

**Published:** 2022-11-11

**Authors:** Isabela Cristina Januário Silva, Marise Lazaretti-Castro

**Affiliations:** 1 Universidade Federal de São Paulo Departamento de Medicina Disciplina de Endocrinologia São Paulo SP Brasil Disciplina de Endocrinologia, Departamento de Medicina, Universidade Federal de São Paulo (Unifesp), São Paulo, SP, Brasil

**Keywords:** Vitamin D, randomized controlled trial, vitamin D/metabolism, vitamin D/physiology, vitamin D/therapeutic use

## Abstract

Vitamin D deficiency is a general health problem affecting individuals at all stages of life and on different continents. The musculoskeletal effects of vitamin D are well known. Its deficiency causes rickets, osteomalacia, and secondary hyperparathyroidism and increases the risk of fractures. Clinical and experimental evidence suggests that vitamin D performs multiple extraskeletal functions. Several tissues unrelated to calcium and phosphate metabolism express vitamin D receptor (VDR) and are directly or indirectly influenced by 1,25(OH)_2_D (calcitriol). Some also express the enzyme 1 alpha-hydroxylase (CYP27B1) and produce 1,25(OH)_2_D, inducing autocrine or paracrine effects. Among the pleiotropic effects of vitamin D are the regulation of cell proliferation and differentiation, hormone secretion, and immune function. In this review, we outline vitamin D physiology and the outcomes of recent large RCTs on its potential extraskeletal effects. Those studies exhibit a need for continued clinical analysis to elucidate whether vitamin D status can influence extraskeletal health. Longer longitudinal follow-up and standardized assays are crucial to better assess potential outcomes.

## INTRODUCTION

Vitamin D photosynthesis in tropical countries with low-latitude regions is sufficient in most seasons of the year due to the wavelength of UV-B rays. Nevertheless, studies in these countries, including Brazil, have reported a high prevalence of vitamin D deficiency (levels of 25-hydroxyvitamin D < 20 ng/mL) in various age groups, similarly to other populations worldwide (1,2). Therefore, vitamin D deficiency is a general health problem, as it affects individuals at all stages of life and on different continents (1,3).

Serum 25-hydroxyvitamin D (25OHD) levels are used to assess vitamin D status. Deficiency is defined as levels below 20 ng/mL, and severe deficiency is defined as levels below 12 ng/mL; the latter strongly increases the risk of rickets and osteomalacia. Values between 20 and 60 ng/mL are considered adequate for the general population under 65 years, and levels between 30 and 60 ng/mL are recommended for individuals in vulnerable conditions. Table 1 describes high-risk individuals for whom levels above 30 ng/mL are recommended. The risk of intoxication with hypercalcemia and its repercussions increases considerably with levels above 100 ng/mL, but exceeding 60 ng/mL is not recommended due to the lack of evidence of any benefit (4,5).

**Table 1:** Clinical conditions in which patients could benefit from 25-hydroxyvitamin concentrations above 30 ng/mL

Vulnerable conditions
Elderly (>65 years)
Pregnant women
Osteoporosis (primary or secondary)
Fractures due to fragility
Metabolic bone diseases*
Secondary hyperparathyroidism
Sarcopenia
Recurring falls
Chronic renal disease
Malabsorption syndrome
Liver failure
Type 1 Diabetes mellitus
Cancer

* Osteomalacia, osteogenesis imperfecta, primary hyperparathyroidism.

Adapted from: Moreira and cols.(5).

Although skin synthesis occurs rapidly after sun exposure, vitamin D status is influenced by several environmental factors. Among these, low exposure to UVB radiation stands out; this factor is partly dependent on latitude, the season of the year, and the degree of air pollution. The following factors may also interfere with vitamin D status: low intake of foods rich in vitamin D, skin aging, high body mass index, intestinal microbiota changes, heavier clothing, higher skin phototypes, and genetic factors of some ethnicities (6–8). Mendelian randomizations have shown that single nucleotide polymorphisms (SNPs) can explain 2%-10% of variations in 25OHD levels (9–11).

In a meta-analysis involving 340,476 Brazilians, the prevalence of vitamin D deficiency was 28.16% (95% CI: 23.90, 32.40) and the prevalence of insufficiency (25OHD < 30 ng/mL) was 45.26% (95% CI: 35.82, 54.71). However, most of the population of the analyzed studies consisted of elderly and postmenopausal women (1). Furthermore, awareness regarding the risk of skin cancer and, consequently, the encouragement of sunscreen use may have interfered with the prevalence of vitamin D deficiency. Sun protection factor 8 reduces vitamin D photoproduction by 90%, and sun protection factor 30 reduces it by 99% (2).

The musculoskeletal effects of vitamin D are well known. Its deficiency causes rickets, osteomalacia, and secondary hyperparathyroidism and increases the risk of fractures (12). Furthermore, clinical, and experimental evidence suggests that vitamin D performs multiple extraskeletal functions.

Several tissues unrelated to calcium and phosphate metabolism express vitamin D receptor (VDR) and are directly or indirectly influenced by 1,25(OH)_2_D. Some express the enzyme 1 alpha-hydroxylase (CYP27B1), which is responsible for the synthesis of the active form of vitamin D (1,25(OH)_2_D), with autocrine or paracrine action (6). Among the pleiotropic effects of vitamin D are the regulation of cell proliferation and differentiation, hormone secretion, and immune function (7,13). In this review we summarize the physiology of vitamin D and the results of recent large RCTs about its potential extraskeletal effects.

## PHYSIOLOGY

Most of the circulating vitamin D metabolites come from cutaneous photosynthesis. Solar radiation enables the conversion of 7-dehydrocholesterol (7-DHC) into pre-vitamin D3, which is thermally isomerized into vitamin D3 (cholecalciferol) (13,14).

To a lesser extent than cutaneous photosynthesis vitamin D2 (ergocalciferol) and D3 contained in egg yolks, dairy products, fortified cereals, some fat fish, and sun-dried mushrooms are absorbed in the lumen of the small intestine. After intestinal absorption, vitamin D binds to chylomicrons and is transported from the lymphatic vessels into systemic circulation (6–8).

All vitamin D metabolites are lipophilic and must be transported through plasma bound to a vitamin D binding protein (VDBP) and, in smaller proportions, to albumin. Vitamin D2 and D3 are then transported to the liver, where they are hydroxylated by the enzyme 25-hydroxylase (CYP2R1) into 25-hydroxyvitamin D or 25OHD (calcidiol). Thus, 25OHD is the circulating storage form of vitamin D, which mainly accumulates in adipose tissue and skeletal muscle and is capable of maintaining adequate levels for a few months even in the absence of ingestion or when exposed to UV-B radiation (2,15). The 2-to-3-week half-life of 25OHD ensures lower serum fluctuation and reflects both cutaneous synthesis and diet contributions. For now, it is considered the best indicator of vitamin D sufficiency (8,11,16).

**Figure 1:**
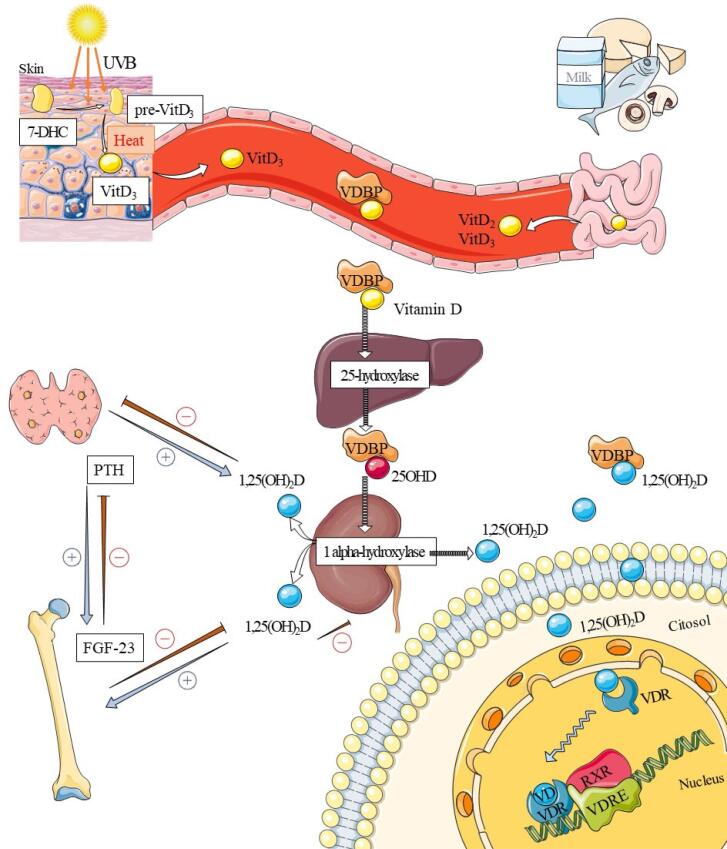
Basic physiology of vitamin D action. Solar radiation converts 7-dehydrocholesterol (7-DHC) into pre-vitamin D (pre-vit D_3_) and thermo-isomerize it to vitamin D3 (VitD_3_). Some foods are a source of vitamin D2 and D3 in smaller amounts. Vitamin D (VD) is transported through the plasma by vitamin D binding protein (VDBP), and undergoes two hydroxylations; in the liver, it is converted by the enzyme 25-hydroxylase into 25-hydroxyvitamin D (25OHD), and in the kidneys, the enzyme 1 alpha-hydroxylase converts it into 1,25-dihydroxyvitamin D (1,25(OH)_2_D), the active form of vitamin D. When binding to the vitamin D receptor (VDR), it undergoes heterodimerization with the retinoic acid receptor (RXR) in the cell nucleus. This heterodimer binds to the vitamin D responsive element (VDRE), culminating in the regulation and expression of several genes. Among its roles, it inhibits PTH synthesis and stimulates the gene expression of FGF-23. This representation was partly generated using Servier Medical Art, provided by Servier, licensed under a Creative Commons Attribution 3.0 unported license.

25OHD is transported to the renal circulation as a 25OHD-VDBP complex, where it is filtered by the glomeruli. The megalin/cubulin membrane receptors expressed in renal tubular cells help with the endocytic internalization of the 25OHD-VDBP complex, preventing its urinary excretion (14,17). Under the actions of the enzyme 1 alpha-hydroxylase (CYP27B1) expressed in tubular cells, 1,25-dihydroxyvitamin D (1,25(OH)_2_D; also called calcitriol) is produced (Figure 1), which is the biologically active form with 6-8 hours of half-life. With sufficient vitamin D, the precursor 25OHD and 1,25(OH)_2_D can be hydroxylated to inactive metabolites by 24-hydroxylases (CYP24A1) and excreted through bile, feces, and urine (4,7,8).

The activity of 1 alpha-hydroxylase determines the amount of circulating active vitamin D; PTH is one of its most important stimulating factors. In the face of hypocalcemia, calcium sensor-receptors (CaSR) in the parathyroid cells induce PTH synthesis, which stimulates the *CYP27B1* gene encoding 1 alpha-hydroxylase and synthesizes 1,25(OH)_2_D. The latter provides negative feedback, inhibiting the *CYP27B1* and decreasing PTH levels, directly suppressing its gene transcription from parathyroids and indirectly suppressing it by increasing calcemia (8,18).

Vitamin D receptors (VDR) are nuclear receptors that are widely expressed, even in tissues not associated with calcium and phosphorus transportation. 1,25(OH)2D has a high affinity for VDR. After binding, there is a conformational change of receptor and heterodimerization with the retinoic acid receptor (RXR). This ligand-VDR/RXR complex couples to sites known as vitamin D responsive elements (VDRE) present in the promoter region of target genes. As a result, gene transcription is regulated in various tissues with co-activators or corepressors. Translation occurs after gene transcription, resulting in the formation of proteins involved in, for instance, transcellular calcium transport (8,14,18).

1,25(OH)_2_D/VDR-RXR acts primarily on small intestinal enterocytes, inducing the expression of receptors that mediate intestinal calcium absorption, such as TRPV6, CaBP9k, PMCA1b, and CLDN2. In the parathyroids, this complex increases the expression and sensitivity of CaSRs. In the kidneys, it induces the transcription of some genes, such as *Klotho* (KL), the co-receptor for the FGF-23/FGFR1 signaling; NPT2a and NPT2c, the channels responsible for renal phosphate reabsorption and correction of hypophosphatemia; and TRPV5 and CaBP28k, which increase renal calcium reabsorption. It downregulated its own production, stimulating 24-hydroxylase while suppressing 1 alpha-hydroxylase and consequently decreasing circulating 1,25(OH)_2_D. 1,25(OH)_2_D/VDR-RXR also induces the transcription of various bone-related genes, such as *RANKL*, which promotes osteoclastogenesis and bone resorption, and *SPP1*, which encodes the crucial protein osteopontin (OPN). OPN binds to αvβ3-integrin and activates a Pi3K/AKT pathway to synthetize another protein, the MZF1 transcriptional factor, which directly stimulates FGF-23 transcription (14,18).

**Figure 2:**
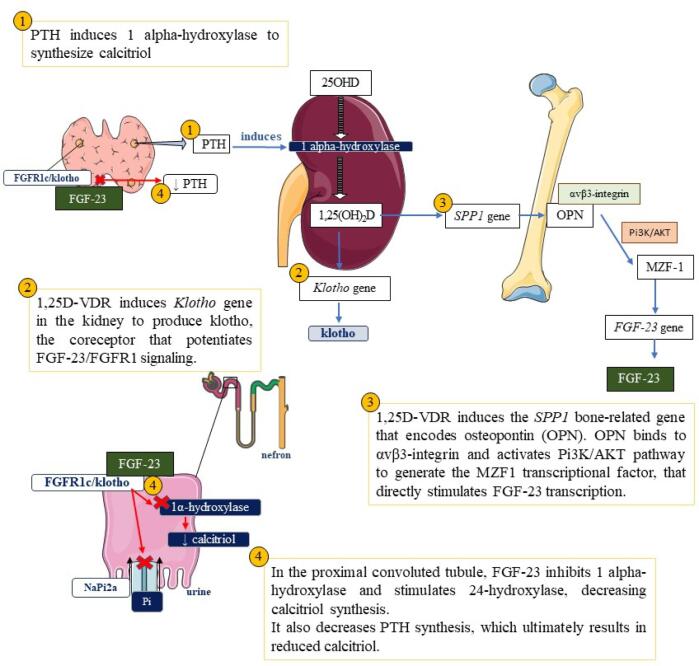
Model for Secondary Regulation of Fibroblast Growth Factor-23 by 1,25(OH)_2_D/VDR. (Amended from Haussler et al., 2021) (18). [This representation was generated using Servier Medical Art, provided by Servier, licensed under a Creative Commons Attribution 3.0 unported license (https://creativecommons.org/licenses/by/3.0/)].

FGF23 is a major phosphate-regulating hormone known as “phosphatonin,” produced by osteocytes and osteoblasts. Its production is induced by increased 1,25(OH)_2_D, PTH, hyperphosphatemia, hypercalcemia, inflammation, and hypoferremia. After its skeletal production, FGF-23 binds to the renal receptors FGFR1c and Klotho to promote phosphaturia while maintaining serum calcium levels by stimulating renal reabsorption. It also plays an essential role in vitamin D metabolism because it inhibits 1,25(OH)_2_D synthesis by suppressing *CYP27B1* and increases its catabolism by inducing *CYP24A1*. The physiologic role of FGF-23 is to limit excessive bone mineralization by decreasing serum phosphate and 1,25(OH)_2_D levels (Figure 2) (18).

## MAJOR EXTRASKELETAL OUTCOMES

Several large randomized clinical trials focusing on the extraskeletal actions of vitamin D have been developed in recent years. Table 2 summarizes the main characteristics of the latest studies; their main results will be detailed below.

**Table 2:** Overview of recent randomized double-blind clinical trials about potential extraskeletal benefits of vitamin D supplementation

Study	N° of individuals	Years of follow-up (mean)	Interventions	Primary outcomes	Summary of results	Post-hoc analysis
VITAL (25) (USA)	25,874	5.3	Vitamin D3 at a dose of 2,000 IU/day; omega-3 at a dose of1 g/day	Cancer and CV disease	Supplementation with vitamin D did not result in lower incidents of invasive cancer or CV events than placebo	Significant reduction in cancer mortality in the supplementation group after excluding deaths in the first year
				Ancillary study of autoimmune diseases	Resulted in lower incidents of autoimmune diseases than placebo group	Over the last 3 years of intervention, the supplemented group had 39% fewer participants with confirmed autoimmune diseases
ViDA (26)(New Zealand)	5,110	3.3	One dose of 200,000and 100,000 IU/month	CV events and mortality	Vitamin D supplementation does not decrease the risk of cardiovascular disease	Vitamin D supplementation did not reduce cancer incidences
D2d (27)(USA)	2,423	2.5	4,000 IU/day	T2DM	No effect in progression of prediabetes into T2DM	Significant reduction in progression into T2DM in individuals with baseline BMI < 30 kg/m^2^, severe vitamin D deficiency, good adherence, or serum 25OHD > 40 ng/mL
DO-HEALTH (24)(Europe)	2,157	3	2,000 IU/day; omega 3 at 1 g/day;strength-training exerciseprogram	Systolic and diastolic blood pressure, physical and cognitive performance, non-vertebral fractures and infections	Overall, there were no statistically significant benefits of any intervention, individually or combined, for the 6 endpoints after 3 years	Subgroup comparisons had statistically significant results, but should be considered for hypothesis generation due to the main null effect and the large number of statistical comparisons
FIND (23)(Finland)	2,495	5.0	1,600 IU/day or 3,200 IU/day	Incident of major CVD and invasive cancer	Supplementation did not reduce the incidents of major CVD events, invasive cancer, or mortality among generally healthy and mostly vitamin D-sufficient older adults in Finland	

### Cancer

The potential benefits of vitamin D supplementation in preventing cancer were initially suggested based on ecological and observational studies that demonstrated reduced cancer mortality in areas with higher sun exposure compared to areas with low sun exposure (19,20). In addition, experimental studies demonstrated that vitamin D metabolites affect cell differentiation, inhibit cancer cell proliferation, and generate anti-inflammatory and immunomodulatory effects. These observations gave plausibility to the possible anti-neoplastic role of vitamin D, which many clinical trials aimed to investigate (10).

The VITAL trial, one of the most recent and, so far, the largest trial, evaluated over 25,000 American adults and found no effect on incidents of invasive cancer from 2,000 IU daily vitamin D supplementation (HR 0.96, 95% CI 0.88-1.06) compared to placebo in 5.3 years of follow-up. The sub-analyses suggested a trend towards reduced cancer risk in individuals with a BMI < 25 kg/m^2^ and in African-Americans. Post-hoc analyses that excluded deaths in the first year of randomization demonstrated a statistically significant reduction in cancer mortality in the vitamin D-supplemented group (HR 0.75, 95% CI 0.59-0.96). After four years, it was possible to assess the dissociation of the mortality curve between the supplemented vs. non-supplemented groups through a Kaplan-Meier plot (10,20,21). One of the most important limitations was the mean of 25OHD concentrations at baseline, which was already high, with few participants with deficiency. Individuals in the placebo group were allowed to take up to 800 IU of vitamin D daily.

In the ViDA trial, New Zealand adults were supplemented with 100,000 IU of vitamin D or placebo per month for 3.3 years with no differences in cancer outcomes. The study duration may have been one of the limitations of the work because the effect of supplementation was only confirmed after four years in the VITAL trial. In addition, whether intermittent bolus doses could cause non-physiological fluctuations in vitamin D and interfere with outcomes is questionable (10,22).

In the randomized double-blind placebo-controlled FIND study, 2,495 healthy participants were randomized to receive 1,600 IU/day, 3,200 IU/day, or placebo and were followed for five years. One of the primary outcomes was incidents of invasive cancer or cancer outcomes, which were not reduced with supplementation. One of the study's limitations was how few participants had vitamin D insufficiency, possibly due to Finland's national food fortification policies. Tracking incident events will continue for years and may provide more relevant information (23).

These studies contributed to elaborating the methodology of in-progress interventionist studies. In addition, Mendelian randomizations may help understand which individuals would benefit from vitamin D supplementation (21).

### Cardiovascular diseases

The causal relationship between vitamin D and cardiovascular disease was investigated because preclinical studies documented the presence of VDR in endothelial cells and cardiomyocytes. These laboratory and animal studies demonstrated that VDR knocks out mice who developed hypertension and found that vitamin D helps regulate the renin-angiotensin-aldosterone system by suppressing renin gene expression (24).

However, two significant RCTs (VITAL and ViDA) that were designed to include cardiovascular events in their primary endpoints found no benefit from supplementation. Among the limitations of the two studies was the small number of participants with severe vitamin D deficiency (10).

During the 5.3 years of VITAL, the HR for MACE (myocardial infarct, stroke, and cardiovascular mortality) was 0.97 (95% CI 0.85-1.12) in the supplemented group. There was no difference in outcomes after excluding events from the first two years (21). The ViDA study evaluated MACE as a primary endpoint through monthly 100,000 IU vitamin D supplementation over three years and found no benefit (HR 1.02, 95% CI 0.87-1.20) (10). Another recent trial, DO-HEALTH, was conducted in 5 European countries among 2,157 adults with a median age of 70 years and showed no improvement in systolic and diastolic blood pressure, one of the primary evaluated endpoints (24). The Finns trial (FIND) that was cited earlier also evaluated the primary outcome of CVD incidences and found null results (23). Meta-analyses of several RCTs, including VITAL and ViDA, obtained similar results (10).

### Type 2 diabetes mellitus

The hypotheses of the influence of vitamin D on the risk of progression from prediabetes to Type 2 diabetes mellitus (T2DM) were based on *in vivo* and *in vitro* studies that demonstrated the capability of pancreatic Beta-cells to synthesize VDR and 1 alpha-hydroxylase, thus producing 1,25(OH)2D locally. In these experimental studies, active vitamin D was capable of modulating the function of pancreatic beta cells and improving insulin sensitivity (28). Furthermore, they found that vitamin D deficiency in rats leads to reduced insulin secretion, which is restored after its supplementation (29).

Following this rational, the D2d randomized controlled trial was designed to investigate whether vitamin D supplementation could decrease prediabetes transformation into type 2 diabetes. It included 2,423 participants with prediabetes who, after 2.5 years of follow up, showed a non-significant downward trend in progression to T2DM in groups who received vitamin D (HR 0.88, CI 0.75-1.04, *P* = 0.12) (10). A small subgroup without obesity had modest benefits, but these were less than those obtained through lifestyle changes and metformin use. The restricted benefit for participants with obesity may relate to reduced 25-alpha-hydroxylase activity and sequesters of vitamin D on adipose tissue, resulting in the need for higher doses to reach the same serum concentrations (28).

Post-hoc analyses of the D2d study supported the modest but significant effect of supplementation in subjects with prediabetes, particularly those with baseline BMI < 30 kg/m^2^, baseline vitamin D deficiency, good adherence, and maintenance of serum levels above 40 ng/mL for most of the follow-up. Participants who maintained 25OHD levels during the trial with 50 ng/mL or more had a more significant risk reduction (HR 0.29, CI 0.17-0.50), while those with levels between 40-49 ng/mL had a partial risk reduction (HR 0.48, CI 0.29-0.80) for progression to T2DM compared to participants who maintained levels between 20-29 ng/mL. One of the study's limitations that may have reduced the effect was the small number of participants with vitamin D deficiency at baseline (10,27,28).

Analysis of combined data from D2d and two other trials demonstrated potential risk reduction for developing T2DM. However, Mendelian randomizations did not support these conclusions (10,28).

Due to the global impact of diabetes, even a slight reduction in the risk of progression can have great implications for health policies. Longer follow-up studies are needed to determine whether these benefits are consistent (27,29).

### Autoimmunity

Preclinical studies indicate that 1,25(OH)2D regulates different genes involved in inflammatory responses and innate immunity. The VDR is expressed on dendritic cells, T and B lymphocytes, and macrophages. 1,25(OH)2D suppresses autoantibody production for B cells and T helper-1 lymphocyte-mediated responses, reducing the inflammatory cytokines IL2, interferon-gamma, and tumor necrosis factor (12). When binding to the VDR of CD4+ T cells, it inhibits IL6, which plays an essential role in developing autoimmune diseases, by stimulating T helper lymphocytes 17 (25).

Analogs and 1,25(OH)2D proved effective in treating psoriasis, a disease in which keratinocyte proliferation occurs and has inflammatory and autoimmune components (7). In addition, Mendelian randomizations have provided strong evidence supporting a causal relationship between genetically low levels of 25OHD and increased risk of multiple sclerosis (MS) (10). However, few intervention studies are designed to evaluate these findings, and those already conducted show controverse results (7).

The effects of vitamin D on type 1 diabetes were investigated in an 18-month randomized double-blind placebo-controlled trial where 38 patients newly diagnosed with Type 1 diabetes mellitus (T1DM) were randomly assigned to receive 2,000 IU daily of cholecalciferol or placebo. Treated patients had a significant increase in fasting and stimulated C-peptide levels. The authors concluded that 25OHD supplementation is associated with a protective immunologic effect and a slow decline in residual β-cell function in patients with new-onset T1DM (30).

The VITAL trial conducted to evaluate the role of vitamin D in preventing cancer and cardiovascular disease had an ancillary study initiated prior to recruitment. This study defined incidence of confirmed autoimmune diseases as the primary endpoint. Through annual questionnaires and medical record reviews, new diagnoses of rheumatoid arthritis, polymyalgia rheumatic, autoimmune thyroid disease, psoriasis, and inflammatory bowel disease were evaluated. Over 5 years, daily supplementation with 2,000 IU of vitamin D resulted in lower incidents of autoimmune diseases (HR 0.78, 95% CI 0.61-0.99, *P* = 0.05) than the placebo group. When considering only the last three years of the intervention, the group supplemented with vitamin D had 39% less confirmed autoimmune diseases than the placebo group (*P* = 0.005) (25).

One of the clinical trial limitations was the recruitment of older participants, as autoimmune diseases primarily develop in young adults. Despite this, the data is encouraging and demonstrates that it is a well-tolerated and non-toxic therapy for difficult-to-manage diseases (10,25). Due to the latency of those diagnoses, a long-term follow-up could clarify whether these effects will be potentiated.

In conclusion, vitamin D deficiency remains prevalent worldwide. So far, serum levels of 25OHD < 20 ng/mL are considered deficient for the general population and serum levels of <10 to 12 ng/mL increase the risk of rickets and osteomalacia. Individuals at high-risk for some clinical conditions could benefit from 25OHD concentrations maintained between 30-60 ng/mL (Table 1). Levels above 60 ng/mL are not recommended because of the lack of benefits and higher risk of intoxication.

Observational and experimental data revealed potential extraskeletal effects showing that different tissues unrelated to calcium and phosphorus metabolism express VDR and CYP27B1, which can regulate gene expression in various tissues. Among the new findings, the essential role of FGF-23 in vitamin D metabolism stands out because it limits bone mineralization and reduces circulating 1,25(OH)_2_D by suppressing 1 alpha-hydroxylase.

The recent RCTs aim at extraskeletal effects demonstrated that increasing serum 25OHD in replete individuals does not generate benefits for preventing major diseases such as cancer, T2DM, and autoimmune and cardiovascular diseases. Regardless, the subgroup or post-hoc analyses suggested promising effects in reducing progression to T2DM, decreasing cancer mortality, and decreasing incidence of autoimmune disease.

Indeed, these findings not only brought some enthusiasm, but also provided information on the need for more appropriate study designs and longer follow-up periods to reach more solid conclusions on the extraskeletal effects of vitamin D supplementation.
